# DNA: Novel Crystallization Regulator for Solid Polymer Electrolytes in High-Performance Lithium-Ion Batteries

**DOI:** 10.3390/nano14201670

**Published:** 2024-10-17

**Authors:** Xiong Cheng, Joonho Bae

**Affiliations:** Department of Physics, Gachon University, Seongnam-si 13120, Gyeonggi-do, Republic of Korea

**Keywords:** DNA, polyvinylidene fluoride (PVDF), solid polymer electrolytes

## Abstract

In this work, we designed a novel polyvinylidene fluoride (PVDF)@DNA solid polymer electrolyte, wherein DNA, as a plasticizer-like additive, reduced the crystallinity of the solid polymer electrolyte and improved its ionic conductivity. At the same time, due to its Lewis acid effect, DNA promotes the dissociation of lithium salts when interacting with lithium salt anions and can also fix the anions, creating more free lithium ions in the electrolyte and thus improving its ionic conductivity. However, owing to hydrogen bonding between DNA and PVDF, excess DNA occupies the lone pairs of electrons of the fluorine atoms on the PVDF molecular chains, affecting the conduction of lithium ions and the conductivity of the solid electrolyte. Hence, in this study, we investigated the effects of adding different DNA amounts to solid polymer electrolytes. The results show that 1% DNA addition resulted in the best improvement in the electrochemical performance of the electrolyte, demonstrating a high ionic conductivity of 3.74 × 10^−5^ S/cm (25 °C). The initial capacity reached 120 mAh/g; moreover, after 500 cycles, the all-solid-state batteries exhibited a capacity retention of approximately 71%, showing an outstanding cycling performance.

## 1. Introduction

Lithium-ion batteries have been extensively utilized in modern applications owing to their remarkable energy density and extended service life [1,2,3]. However, prevalent safety concerns, such as electrolyte leakage [4] and separator punctures leading to short circuits [5], have led to the development of solid electrolytes for next-generation lithium batteries [6,7]. All-solid-state lithium batteries are primarily categorized into three types: inorganic solid electrolytes (ISEs) [8], solid polymer electrolytes (SPEs) [9], and composite polymer electrolytes (CPEs) [10]. Solid polymer electrolytes have garnered attention from numerous research institutions owing to their affordability, superior stability, and flexibility [11,12]. Solid polymer electrolytes offer a promising avenue for addressing safety issues while advancing the capabilities of lithium batteries for diverse applications.

Polymers like polyethylene oxide (PEO) [13], polyvinylidene fluoride (PVDF) [14], and polyacrylonitrile (PAN) [15] are commonly utilized in the preparation of solid electrolytes. In particular, PVDF exhibits a remarkable ability to interact weakly with lithium ions [16], attributed to the abundance of highly polar fluorine atoms along its molecular chain, which facilitates the dissociation of lithium salts, thereby augmenting the conductivity of solid electrolytes [17]. The conductivities of PVDF-based solid electrolytes depend predominantly on their amorphous component [18]. Nonetheless, the high symmetry of the PVDF molecular chain makes it prone to crystallization, thereby impeding ion transmission efficiency. Present research endeavors have primarily focused on mitigating the crystallinity of PVDF [19]. Strategies include using PVDF-HFP block copolymers [20] or incorporating specific inorganic conductive fillers such as lithium lanthanum zirconate (LLZO) [21] and lithium lanthanum tungstate (LLZWO) [22], as well as others [23,24]. These approaches aim to disrupt the crystallization process, enhancing the amorphous fraction and, consequently, the ionic conductivity of PVDF-based solid electrolytes [25]. By addressing crystallinity issues, researchers have aimed to exploit the full potential of PVDF-based solid electrolytes, thereby advancing the development of high-performance lithium battery technologies.

DNA, a renewable and environmentally friendly material [26], has emerged as a promising alternative. Its exceptional molecular structure allows precise manipulation at the nanoscale, allowing for the design of customized structures for targeted energy storage applications [27,28]. Incorporating biological macromolecules such as DNA into electrochemical devices offers a significant strategy for advancing sustainable development [29]. Several studies have reported on applying DNA in electrochemical storage devices. For example, Kim et al. [30] presented an innovative binder made from DNA and alginate for silicon and silicon–graphite composite electrodes, thus highlighting the potential of using DNA in lithium-ion battery electrodes. Leones et al. [31] examined DNA-based membranes infused with erbium triflate and showcased high ion conductivity and redox stability suitable for applications in lithium batteries. In a previous study, we successfully integrated DNA with carbon nanotubes to produce nickel–cobalt oxide DNA composite materials, demonstrating their potential in supercapacitors [32]. Additionally, we utilized DNA composite materials in lithium battery separators, which enhanced lithium–sulfur efficiency and battery stability [33]. These findings underscore the versatility and potential of DNA-based materials for addressing key challenges in energy storage and advancing sustainable technologies. In general, increasing the concentration of polar groups in polymers or decreasing the lattice energy of the added salts leads to a higher concentration of charge carriers [34]. The abundance of polar groups in DNA makes it a promising candidate for enhancing the ionic conductivity of solid electrolytes.

In this study, we introduced DNA into a PVDF polymer electrolyte for the first time, fabricating a novel PVDF@DNA composite solid-state electrolyte. Acting as a plasticizer-like additive, DNA effectively reduces the crystallinity of the solid polymer electrolyte, thereby enhancing its ionic conductivity. Additionally, due to its Lewis acid properties, DNA promotes the dissociation of lithium salts by interacting with lithium salt anions and immobilizing them, which increases the concentration of free lithium ions in the electrolyte, further improving ionic conductivity. The PVDF@1%DNA solid electrolyte demonstrated the highest electrochemical performance, with a capacity of 120 mAh/g at 0.5 C and an impressive service life, retaining ~71% of its capacity after 500 cycles.

## 2. Experimental Methods

### 2.1. Materials

Polyvinylidene fluoride (PVDF), lithium bis (trifluoromethanesulphonyl), imide (LiTFSI), dimethylacetamide (DMAc), lithium iron phosphate (LiFePO_4_, LFP), and salmon deoxyribonucleic acid (DNA) were obtained from Sigma-Aldrich (Darmstadt, Germany). The super-P carbon black was supplied by TIMCAL (Bodio, Switzerland). All chemicals employed in this study were of analytical grade, and used directly without further purification. The detailed information of the materials used in this article is shown in Table S1.

### 2.2. Sample Preparation

**Figure 1:**
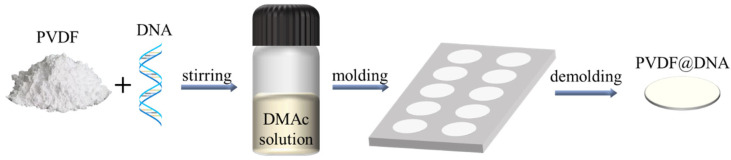
Schematic illustration of preparation of PVDF@DNA solid electrolytes.

PVDF@DNA solid-state electrolytes were successfully prepared by solution blending. In a typical preparation process, 500 mg of PVDF, 500 mg of LiTFSI, and 5 mg of salmon DNA (200 base pairs; Sigma Aldrich) were dissolved in 5 mL of DMAc and stirred at room temperature (25 °C) for 12 h until all bubbles were removed from the solution. Then, the solution was poured into a mold and fully dried at 120 °C to obtain a polymer solid electrolyte with a thickness of approximately 100 μm. Other PVDF@DNA composite solid electrolytes with different DNA weight ratios were prepared using the same method, as shown in Figure 1.

LiFePO_4_ (active material), super-P carbon black (conductive additive), and PVDF (binder) were mixed in N-Methyl-2-Pyrrolidone (NMP) at a mass ratio of 8:1:1 to create a slurry, which was then used to coat aluminum current-collector foil. The coated foil was subsequently dried under vacuum at 80 °C for 12 h and punched into 12 mm disks to serve as the cathodes for coin cells. The anode consisted of lithium metal. The coin cell was assembled by placing the prepared solid electrolyte between the lithium metal anode and the cathode. All assembly processes were conducted in a glove box filled with argon gas.

### 2.3. Characterization

The crystallization kinetics and weight crystallinity were tested using differential scanning calorimetry (DSC, TA Q100, New Castle, DE, USA). Scanning electron microscopy (SEM, Hitachi SU-8600, Tokyo, Japan) with an accelerating voltage of 5.0 kV was used to observe the morphology of the solid electrolytes. The bonding structure of PVDF@DNA solid electrolytes was confirmed by Fourier-transform infrared spectroscopy (FTIR, Thermo Fisher Scientific iS50, Waltham, MA, USA) over a range from 4000 to 500 cm^−1^. Electrochemical impedance spectroscopy (EIS) was performed on a Versastat 4 potentiostat (AMETEK, Inc., Berwyn, PA, USA) at a frequency range of 1 MHz to 0.01 Hz with an amplitude of 5 mV. Cyclic voltammetry (CV) and linear sweep voltammetry (LSV) were conducted on an advanced electrochemical system (CHI604E, Chenhua, Shanghai, China) within a voltage window of 2.5–4.5 V at a scan rate of 0.1 mV s^−1^ and 2.0~6.0 V and at a scan rate of 10 mV s^−1^, respectively. The charge/discharge performance tests were conducted on the electrochemical workstation (WonATech WBCS3000, Seoul, Republic of Korea) in a voltage range from 2.0 to 4.3 V.

## 3. Results and Discussion

**Figure 2:**
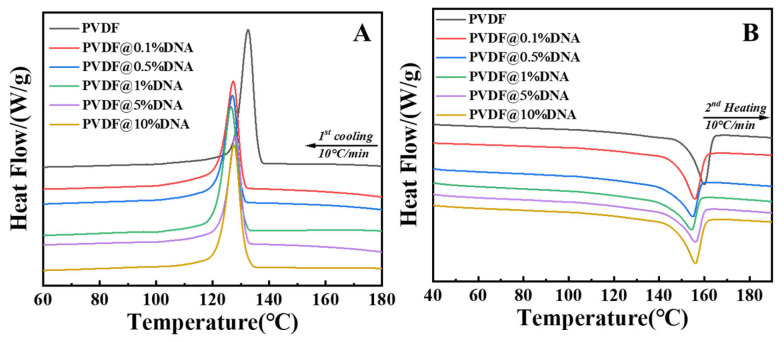
DSC curves of PVDF and PVDF with different ratios of DNA solid polymer electrolytes ((**A**): first cooling process, (**B**): second heating process).

As a low-molecular-weight polymer (Mw ≈ 66,000 g/mol) compared to PVDF (Mw = 534,000 g/mol), DNA is expected to reduce the crystallinity of the PVDF molecular chain during the miscibility process with PVDF, thus increasing its ionic conductivity. DSC measurements were conducted to investigate the compatibility of PVDF and DNA, with the results depicted in Figure 2. As seen from the DSC cooling curve (Figure 2A), adding DNA effectively reduces the crystallization ability of PVDF molecular chain segments, as evidenced by the decreased melt crystallization temperatures of PVDF@DNA during cooling, consistent with the well-known “*T*_m_ depression effect” [35]. The sample with 1% DNA shows the weakest crystallization ability, with a peak located at 126 °C (green curve). The weak crystallization ability clearly decreases the endothermic peaks (melting point) of the solid electrolytes, as observed at 154 °C, consistent with the melting behaviors of PVDF crystals within its blend containing 1 wt.% of DNA; this value notably falls below that of neat PVDF (160 °C, indicated by the black curve). The increase in the melting point and crystallization ability of PVDF@5%DNA and PVDF@10%DNA may be attributed to the entanglement between the molecular chains of DNA and PVDF—equivalent to increasing the molecular chain of PVDF, thus providing more intersections and spaces to form ordered crystalline structures and enhancing its crystallization ability—eventually resulting in an increased melting point and overall crystallinity [36,37]. Calculating the areas of the endothermic peaks indicated that the crystallinity of PVDF decreased from 20% (pure PVDF) to 15% (PVDF@1%DNA); more crystallinity data are provided in the Supporting Information (Table S2). The conduction rate of lithium ions in the amorphous polymer electrolyte surpassed that in the crystalline electrolyte. In other words, the ionic conductivity of the electrolyte increases as the crystallinity of the polymer decreases.

**Figure 3:**
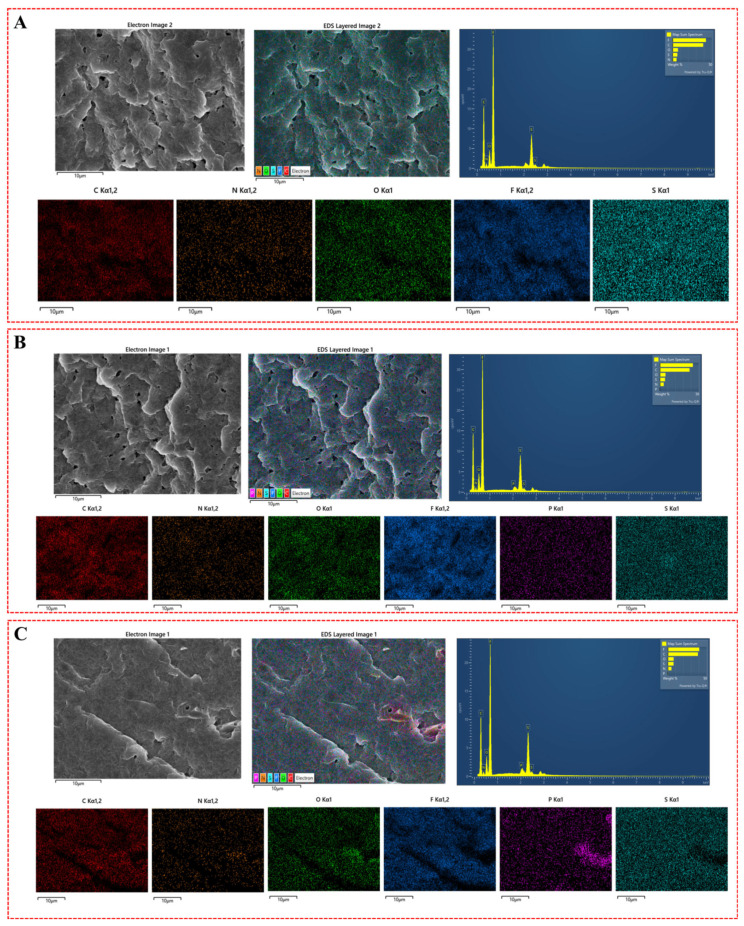
SEM images and EDS mapping of the cross-section of the samples. (**A**): PVDF; (**B**): PVDF@1%DNA; (**C**): PVDF@10%DNA.

The SEM images (Figure 3B,C) of the PVDF@DNA blends’ cross-sections reveal no distinct phase domains, instead displaying a bicontinuous structure across various weight ratios, indicating the absence of phase separation between PVDF and DNA. This homogeneous morphology can be attributed to the thermodynamic compatibility of the PVDF@DNA blend system. DNA, as an amorphous polymer, reduces the crystallinity of PVDF, aligning with typical crystalline/amorphous polymer blend behavior. This compatibility is further confirmed by the absence of two separate glass transition temperatures in the DSC curves (Figure 2), as well as the reduction in melting temperature. The EDS mapping results also support this conclusion. In the pure PVDF electrolyte, the elements are uniformly distributed. However, in the PVDF@1%DNA sample, the appearance of a small amount of phosphorus (from the phosphate groups in DNA) indicates the homogeneous presence of DNA. For the PVDF@10%DNA sample, significant aggregation of the phosphorus element suggests a notable accumulation of DNA at higher concentrations. The SEM and EDS results of other samples are shown in Figure S3. PVDF@5%DNA also showed a certain degree of agglomeration. The presence of a large amount of DNA is entangled with the PVDF molecular chains, which in turn leads to an increase in the overall crystallinity and affects the ionic conductivity of the solid electrolyte. Together, these findings underscore the good compatibility between PVDF and DNA, contributing to the observed uniform structures in both SEM images and DSC results.

**Figure 4:**
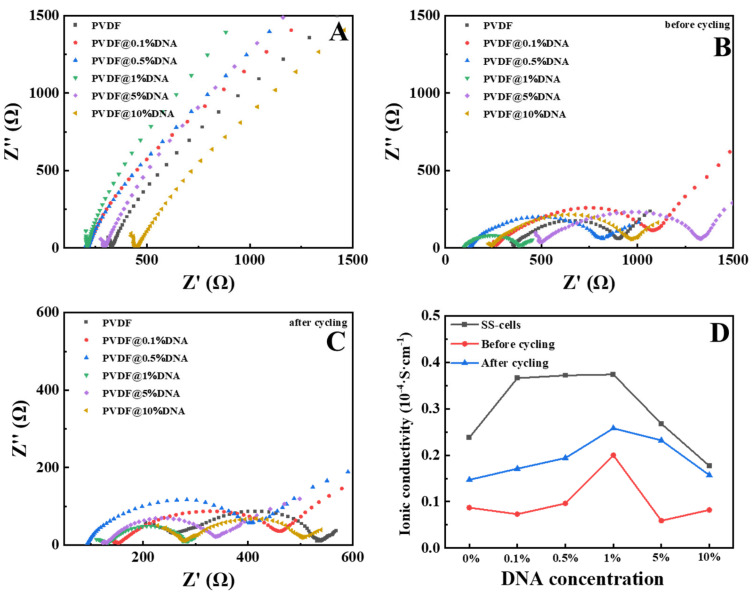
EIS curves of PVDF and PVDF@DNA solid polymer electrolytes in (**A**) SS cells and (**B**,**C**) half-cells. (**D**) Summary of ionic conductivity of all the samples.

Electrochemical impedance spectroscopy (EIS) was performed to test the internal impedances of the polymer electrolytes in SS cells, as displayed in Figure 4A, with most samples containing DNA showing lower impedance than pure PVDF, suggesting that DNA can disrupt the crystallization of PVDF, resulting in increased amorphous area, which facilitates the transport of lithium ions. Additionally, the phosphate groups on the DNA molecular chain are expected to release hydrogen ions, thereby promoting the dissociation of LiTFSI for more free ions and consequently increasing the ionic conductivity of the PVDF@DNA solid electrolyte. EIS spectra revealed that PVDF@1%DNA exhibited a smaller charge-transfer resistance and Warburg impedance than PVDF and PVDF with different ratios of DNA. The corresponding ionic conductivity is measured at 3.74 × 10^−5^ S/cm at room temperature, relatively higher than that of PEO@LiTFSI [38], thus confirming the more efficient charge transfer and faster Li^+^ diffusion kinetics in the solid-state batteries; other ionic conductivity results for the SS cells are presented in the Supporting Information (Table S3). Ionic conduction is primarily influenced by three factors: the inter-chain movement of ions along a chain (τ1), the relaxation of polymer segments (τ2), and intra-chain hopping between different chains (τ3) [39]. The segmental motion of the polymer backbone plays a crucial role in the mobility of both cations and anions. Considering that the PVDF@1%DNA sample showed the lowest crystallinity, it exhibited the lowest impedance.

Furthermore, EIS was employed to elucidate the interfacial reaction resistance, as illustrated in Figure 4B,C. The Nyquist plots for all samples displayed compressed semicircles in the high and mid-frequency regions, representing the charge-transfer resistance (Rct), and sloped lines in the low-frequency region, associated with Warburg impedance (Ws) for lithium-ion diffusion. DNA, a short-chain polar oligomer with a flexible backbone, can form complexes with alkali metal salts, thereby accelerating the local thermal motion and relaxation of polymer segments [40]. The EIS results closely paralleled those of the ionic conductivity tests in the SS cells; compared with pure PVDF, the PVDF@DNA samples showed higher ionic conductivity. Among all the samples, PVDF@1%DNA showed the lowest impedance. Notably, at room temperature, the ionic conductivity measured 2.0 × 10^−5^ S/cm for the 1% DNA sample, while pure PVDF exhibited a higher impedance, correlating to a lower ionic conductivity of 0.87 × 10^−5^ S/cm. After cycling performance tests, the ionic conductivity of PVDF@1%DNA increased to 2.5 × 10^−5^ S/cm, while that of pure PVDF increased to 1.5 × 10^−5^ S/cm; other ionic conductivity results for the half cells are available in the Supporting Information (Tables S4 and S5).

A statistical graph of ionic conductivity is shown in Figure 4D. Notably, the SS exhibited the highest conductivity among the highest cells. Initially, when the battery was assembled, the voltage tended to be unstable and the resistance was relatively high. However, the active material was activated after cyclic testing. Additionally, the chemical compositions of both the positive and negative electrodes gradually transitioned into substances more conducive to electrochemical reactions. This improvement enhances the reaction efficiency of the battery and reduces its internal impedance over time.

**Figure 5:**
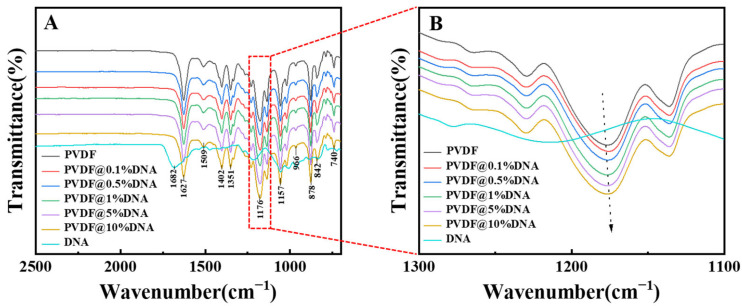
FT-IR curves of solid electrolytes (**A**). An enlarged view of the peak at 1176 cm^−1^ (**B**).

The above analysis concerns the Fourier-transform infrared (FT-IR) spectra of different solid electrolytes. Notably, the characteristic peak of the C-F bond was located at 1176 cm^−1^, as demonstrated in Figure 5A. As the DNA content increased, the peaks broadened and shifted towards lower wavenumbers (Figure 5B), indicating that with higher DNA content, the hydrogen bonds formed between the DNA and PVDF strengthened gradually, leading to a red shift (Figure 5B). Hence, adding DNA disrupts the ordered structure of the PVDF molecular chains and reduces their crystallization ability (Figure 2A), resulting in increased ionic conductivity (Figure 4A). However, owing to the addition of excess DNA, more DNA molecular segments were entangled with PVDF, and hydrogen bonding and entanglement between molecular chains led to an increase in overall crystallinity (Figure 2B, purple curve and yellow curve), which in turn led to a decrease in its ionic conductivity (Figure 4A, purple curve and yellow curve). Therefore, adding 1% DNA ensured both the low crystallinity of PVDF and the mobility of its molecular chains, contributing to the highest ionic conductivity of the PVDF@1%DNA solid polymer electrolyte.

**Figure 6:**
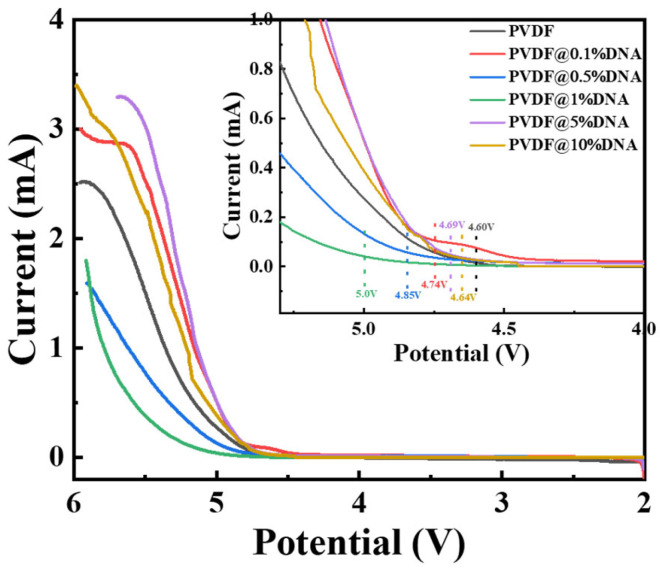
LSV curves of the solid polymer electrolytes in Li-SS cells.

The linear sweep voltammetry (LSV) results for the solid-state electrolyte at room temperature are shown in Figure 6. These results show a consistent pattern across all samples and remain stable in the range of 2–4.3 V, indicating that they can maintain good efficiency during the cycle test. Some samples show an unstable increase in current over 4.6 V, attributed to the decomposition of PVDF when exposed to high-voltage conditions. Upon closer examination, the electrochemical stability windows of different solid-state electrolytes were determined, with the pure PVDF electrolyte exhibiting an electrochemical window of approximately 4.60 V. When DNA was introduced into the PVDF matrix at a concentration of 0.5%, the electrochemical window increased to approximately 4.85 V. Remarkably, the PVDF@1%DNA electrolyte exhibited an even higher electrochemical window, reaching up to approximately 5.0 V. This sequence, in which the electrochemical stability increased from PVDF to PVDF@0.5%DNA and then to PVDF@1%DNA, suggests a clear trend: incorporating DNA into the PVDF matrix significantly enhances the electrochemical stability of the electrolyte. Notably, the electrochemical window of the PVDF@1%DNA electrolyte exceeded 5 V, indicating its superior electrochemical stability within the operational voltage range. This exceptional stability highlights the potential of PVDF@1%DNA as a robust solid-state electrolyte for applications requiring high-voltage performance.

**Figure 7:**
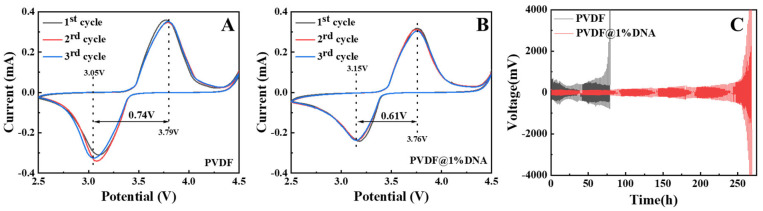
CV curves ((**A**): PVDF, (**B**): PVDF@1%DNA) and (**C**) galvanostatic cycling of Li plating/stripping of Li/SPE/Li symmetrical batteries at room temperature (25 °C), with 0.6 mA·cm^− 2^ current density of solid polymer electrolytes.

**Figure 8:**
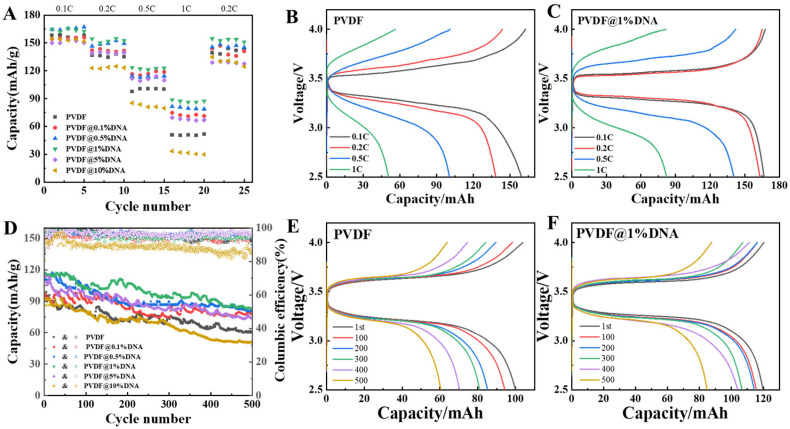
Performance of LiFePO_4_/SPE/Li all-solid-state batteries under 25 °C. (**A**): rate performance; (**B**,**C**): charge–discharge curves of PVDF and PVDF@1%DNA at different C-rates; (**D**): cycling performance; (**E**,**F**): capacity–voltage profiles of PVDF and PVDF@1%DNA at 0.5 C.

CV tests were conducted within the 2.5–4.5 V voltage range at a scan rate of 0.1 mV/s^−1^. Visually, the CV profiles exhibit similar shapes, with reduction peaks observed at approximately 3.1 V and oxidation peaks at approximately 3.8 V. Consequently, the gaps between the oxidation and reduction peaks were calculated. As seen in Figure 7, the gap for pure PVDF (Figure 7A) and PVDF@1%DNA (Figure 7B) was 0.74 V and 0.61 V, respectively; other CV curves are shown in the Supporting Information (Figure S1). PVDF@1%DNA exhibited the narrowest gap between the oxidation and reduction peaks, indicating the least polarization phenomena in the solid-state electrolytes, corresponding to the best stability. Moreover, the peak positions and peak currents of the CV curves for PVDF@1%DNA remained consistent across three cycles, indicating good chemical reversibility of the batteries. Consistent with the redox peaks observed in the CV curves, the charge–discharge curves of LFP display two distinct potential plateaus between 3.1 V and 3.8 V, corresponding to the oxidation of lithium intercalation and deintercalation (as shown in Figure 8B,C).

In the symmetric cells, the symmetrical battery with a pure PVDF solid electrolyte shows a higher start voltage of 650 mV when subjected to an areal capacity of 0.6 mAh·cm^−2^ at room temperature (Figure 7C, black curve). As the system stabilized during the cycle, the voltage gradually decreased to approximately 300 mV. After 23 h of cycling, the voltage gradually increased owing to the continuous growth of lithium dendrites. At approximately 80 h, one side of the puncture by the lithium dendrites caused a short circuit, resulting in a sharp increase in voltage. In contrast, the PVDF@1%DNA solid polymer electrolyte exhibited exceptional performance. Throughout the Li electrodeposition process (Figure 7C), a stable overpotential of approximately 150 mV was maintained with excellent cyclability over an extended cycle time of 100 h without encountering short circuits (Figure 7C, red curve). This stability suggests the effective suppression of Li dendrite growth. At approximately 150 h, the overpotential marginally increased to approximately 200 mV and remained almost constant during subsequent Li electrodeposition for up to 230 h. Other symmetrical battery performance test results are presented in the Supporting Information (Figure S3). The exceptional cyclability observed at room temperature can be attributed to the significantly improved contact with the Li metal anode and the effective suppression of Li dendrite growth during consecutive Li electrodeposition cycles.

To further examine the cycling performance of the coin cells assembled using PVDF and PVDF@DNA solid polymer electrolytes, the relative coin cells were used to evaluate electrochemical performance at various rates (Figure 8A) and to assess cycling performance at a current density of 0.5 C at 25 °C (Figure 8D). The all-solid battery with 1% PVDF DNA showed the highest capacity at a different C-rate (120 mAh/g at 0.5 C) compared with the pure PVDF electrolyte (93 mAh/g at 0.5 C). The corresponding charge–discharge curves are shown in Figure 8B,C, where PVDF@1%DNA always shows a higher capacity than the PVDF electrolyte at every C-rate. Other charge–discharge curves at different C-rates are shown in the Supporting Information (Figure S4). The cell using PVDF@1%DNA as the electrolyte exhibited outstanding cycle stability (Figure 8D); after 500 cycles, a higher value of ~85 mAh/g was retained, indicating ~71% retention. The pure PVDF cells exhibited a capacity of ~60 mAh/g after 500 cycles, with relatively lower stability, indicating that the improved cycling performance of the batteries was due to the combined effects of mechanical properties and ionic conductivity: DNA maintained the stability of the electrolyte by forming hydrogen bonds with the electrolyte while improving the battery capacity by generating more free ions and reducing the crystallinity. With DNA forming hydrogen bonds and preventing the electrolyte from shrinking and deforming, excess DNA resulted in lower capacity due to increased crystallinity. The capacity–voltage profiles of the PVDF and PVDF@DNA cells at different cycles are shown in Figure 8E,F. The cells with PVDF@DNA showed a higher specific capacity and better cycling performance at 0.5 C (1C = 170 mA/g), whereas the capacity of PVDF decreased quickly. Other capacity–voltage profiles are shown in the Supporting Information (Figure S5). In addition, the Coulombic efficiencies of PVDF@DNA were close to 100%, whereas those of PVDF were poor, some even lower than 90% (Figure 8D). Moreover, owing to the presence of DNA, the cell with the PVDF@1%DNA electrolyte delivered a capacity of >115 mAh/g after 100 cycles at the current density of 0.5 C, much higher than that of pure PVDF (~93 mAh/g).

**Figure 9:**
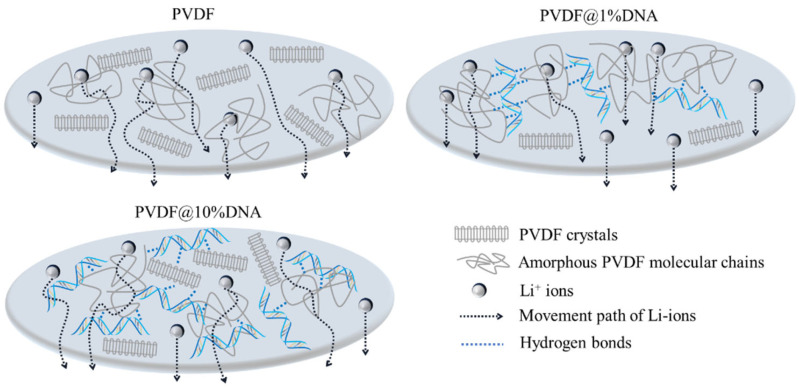
Schematic diagram of the enhanced electrochemical performance mechanism for PVDF@DNA solid polymer electrolytes.

The enhancement of the electrochemical mechanism of the PVDF@DNA is illustrated in Figure 9. Adding DNA at room temperature had a noticeable effect on the crystallization area of the PVDF-based solid polymer electrolyte. In the PVDF@DNA hybrid electrolyte, the amorphous area expanded, facilitating the rapid migration of lithium ions, ultimately leading to enhanced ionic conductivity. In contrast, the structure of the PVDF@DNA electrolyte membranes remained stable during long-term cycling owing to the entanglement between the DNA and PVDF molecular chains. In comparison, pure PVDF electrolyte membranes are prone to shrinkage, deformation, and potential collapse during cycling, resulting in various electrochemical performance issues. This susceptibility to degradation can cause the premature failure of PVDF-based cells, potentially due to capacity decay from short circuits. Notably, the additional DNA in PVDF@DNA can form more hydrogen bonds with PVDF, as depicted in Figure 5; a certain extent of entanglement between DNA and PVDF could promote the transportation of lithium ions because the crystallization ability of PVDF molecular chains is reduced. Thus, by taking advantage of the enhanced Li^+^ ion migration facilitated by DNA, batteries with the PVDF@1%DNA solid polymer electrolyte exhibited excellent electrochemical performance. However, excess DNA affected the transportation of Li^+^ ions owing to the large number of hydrogen bonds and higher crystallinity. The transfer of Li^+^ ions in polymers relies primarily on the coordination and dissociation of polar atoms in amorphous molecular chains. [41] As a result, the migration channels for Li^+^ ions were relatively fewer and longer in PVDF@10%DNA than in PVDF@1%DNA, owing to the occupation of F atoms and entanglements between molecular chains. This delicate balance between enhancing ionic conductivity and reducing migration channels contributes to batteries using the PVDF@1%DNA electrolyte, which exhibits excellent electrochemical performance.

## 4. Conclusions

We fabricated a PVDF@DNA solid polymer electrolyte and found that the incorporation of DNA reduced the crystallinity of PVDF, facilitating the release of more Li^+^ ions and enhancing the ionic conductivity of the electrolyte. Batteries utilizing these DNA-based electrolytes exhibited superior electrochemical performance compared to those with pure PVDF electrolytes. An optimal DNA addition lowered the crystallinity of PVDF and increased ionic conductivity, thus improving the electrochemical performance. However, excessive DNA content raised the crystallinity and blocked more ion transport channels, leading to diminished electrochemical performance. The PVDF@1% DNA sample demonstrated the best performance, with a high capacity of 120 mAh/g at 0.5 C and excellent cycle stability, retaining ~71% of its capacity after 500 cycles.

## Data Availability

The data that support the findings of this study are available on request from the corresponding author upon reasonable request.

## Supplementary Materials

The following supporting information can be downloaded at: https://www.mdpi.com/article/10.3390/nano14201670/s1, Table S1. Specification of materials adopted in this work; Table S2. Calculated percentages of crystallinity of the solid polymer electrolytes; Table S3. Calculated conductivity for solid polymer electrolytes in SS-cells; Table S4. Calculated conductivity for solid electrolytes in half cells before cycling; Table S5. Calculated conductivity for solid electrolytes in half cells after cycling; Figure S1. SEM images and EDS mapping of the cross-section of the samples A: PVDF@ 0.1%; B: PVDF@0.5%DNA; C: PVDF@5%DNA; Figure S2. CV curves of solid polymer electrolytes; Figure S3. Galvanostatic cycling (C) of Li plating/stripping of Li/SPE/Li symmetrical batteries at room temperature (25 °C), with 0.6 mA·cm^−2^ current density of solid polymer electrolytes; Figure S4. The charge-discharge curves for LiFePO4/SPE/Li all-solid-state batteries under different C-rate; Figure S5. The capacity-voltage profiles for LiFePO4/SPE/Li all-solid-state batteries under 0.5 C.
