# Supplementary material for: DNA: Novel Crystallization Regulator for Solid Polymer Electrolytes in High-Performance Lithium-Ion Batteries
Source: Nanomaterials (Basel). 2024 Oct 17;14(20):1670. doi: 10.3390/nano14201670 (PMC11510397; doi:10.3390/nano14201670)
Supplement: Supplementary file 1 [file nanomaterials-14-01670-s001.zip › nanomaterials-3239820-supplementary.pdf]

SUPPORTING INFORMATION

For

DNA：Novel Crystallization Regulator for Solid Polymer Electrolytes in High Performance Lithium-ion Batteries

Xiong Cheng, Man Li, Joonho Bae*

Department of Physics, Gachon University, Seongnam-si, Gyeonggi-do, 13120, Republic of Korea

* Corresponding author: Dr. Bae. J. (baejh2k@gachon.ac.kr, Tel/Fax: +82 31 750 5612)

This Supporting Information file includes:

Tables

		Page Number
**Tab. S1:**	Specification of materials adopted in this work.	S3
**Tab. S2:**	Calculated percentages of crystallinity of the solid polymer electrolytes.temperature (25°C), with 0.6 $mA\cdot cm^{-2}$ current density of solid polymer electrolytes	S3
**Tab. S3:**	Calculated conductivity for solid polymer electrolytes in SS-cells	S4
**Tab. S4:**	Calculated conductivity for solid electrolytes in half cells before cycling	S4
**Tab. S5:**	Calculated conductivity for solid electrolytes in half cells after cycling	S4

Figures

		Page Number
**Fig. S1:**	SEM images and EDS mapping of the cross-section of the samples A: PVDF@ 0.1%; B: PVDF@0.5%DNA; C: PVDF@5%DNA	S5
**Fig. S2:**	CV curves of solid polymer electrolytes	S6
**Fig. S3:**	Galvanostatic cycling (C) of Li plating/stripping of Li/SPE/Li symmetrical batteries at room temperature (25°C), with 0.6 $mA\cdot cm^{-2}$ current density of solid polymer electrolytes	S6
**Fig. S4:**	The charge-discharge curves for $LiFePO_4$/SPE/Li all-solid-state batteries under different C-rate.	S7
**Fig. S5:**	The capacity-voltage profiles for $LiFePO_4$/SPE/Li all-solid-state batteries under 0.5C	S7

Table S1. Specification of materials adopted in this work.

Name	Model	Company
Polyvinylidene fluoride (PVDF)	182702-250G	Sigma-Aldrich
Lithium bis(trifluoromethanesulphonyl) imide (LiTFSI)	544094-100G	Sigma-Aldrich
Dimethylacetamide (DMAc)	270555-1L	Sigma-Aldrich
Lithium iron phosphate ($LiFePO_4$, LFP)	759546-5G	Sigma-Aldrich
Salmon deoxyribonucleic acid (DNA)	31149-10G-F	Sigma-Aldrich
Super-P carbon black	130318	TIMCAL

Calculation of Crystallinity

The DSC curve of the electrolytes was analyzed and the percentage of crystallinity (χ_c) was calculated using the following equation:

$$\chi_c = \frac{\Delta H_m}{\Delta H^*} \times 100\%$$

where, ΔH_m was the enthalpy of fusion of the sample's electrolyte, ΔH^* was the apparent melting enthalpy of a fully crystallized PVDF i.e. 104.7 J g^{-1}.

Employing the aforementioned equation, the resultant crystallinity values were derived as follows.

Table S2. Calculated percentages of crystallinity of the solid polymer electrolytes.

**Sample**	T_m	**Melting enthalpy**	**Crystallinity/%**
PVDF	160.02°C	20.3J/g	19.4
PVDF@0.1%DNA	155.87°C	16.3J/g	15.6
PVDF@0.5%DNA	154.94°C	16.1J/g	15.4
PVDF@1%DNA	154.36°C	15.8J/g	15.1
PVDF@5%DNA	156.07°C	16.5J/g	15.8
PVDF@10%DNA	156.01°C	17.2J/g	16.4

Conductivity of solid polymer electrolytes

Ionic conductivity (σ) of solid polymer electrolytes are calculated using the following equation:

$$\sigma = \frac{L}{RS}$$

where, L, R and S represents the thickness, resistance, and area of the samples respectively.

Table S3. Calculated conductivity for solid polymer electrolytes in SS-cells

Sample	R/Ω	d/cm	S/cm^2	$\sigma/10^{-4}\cdot S\cdot cm^{-1}$
PVDF	330	0.02	2.545	0.238
PVDF@0.1%DNA	215	0.02	2.545	0.366
PVDF@0.5%DNA	211	0.02	2.545	0.372
PVDF@1%DNA	210	0.02	2.545	0.374
PVDF@5%DNA	293	0.02	2.545	0.268
PVDF@10%DNA	445	0.02	2.545	0.177

Table S4. Calculated conductivity for solid electrolytes in half cells before cycling

Sample	R/Ω	d/cm	S/cm^2	$\sigma/10^{-4}\cdot S\cdot cm^{-1}$
PVDF	904.5	0.02	2.545	0.087
PVDF@0.1%DNA	1082	0.02	2.545	0.073
PVDF@0.5%DNA	819.5	0.02	2.545	0.096
PVDF@1%DNA	384.6	0.02	2.545	0.20
PVDF@5%DNA	1331	0.02	2.545	0.059
PVDF@10%DNA	964.2	0.02	2.545	0.082

Table S5. Calculated conductivity for solid electrolytes in half cells after cycling

Sample	R/Ω	d/cm	S/cm^2	$\sigma/10^{-4}\cdot S\cdot cm^{-1}$
PVDF	533.1	0.02	2.545	0.147
PVDF@0.1%DNA	460	0.02	2.545	0.171
PVDF@0.5%DNA	405.2	0.02	2.545	0.194
PVDF@1%DNA	285	0.02	2.545	0.258
PVDF@5%DNA	338.2	0.02	2.545	0.232
PVDF@10%DNA	501.4	0.02	2.545	0.157

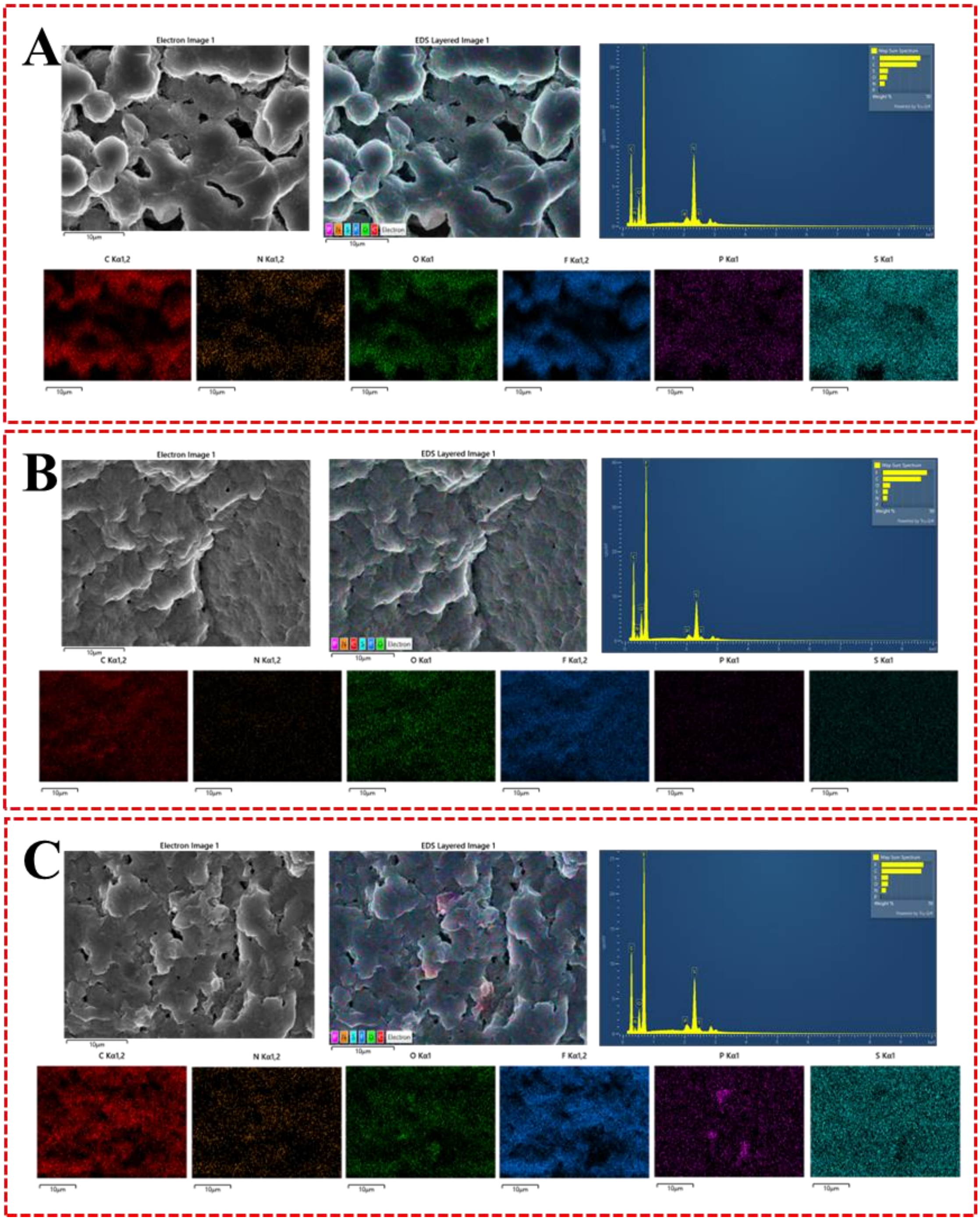

Figure S1. SEM images and EDS mapping of the cross-section of the samples.
A: PVDF@ 0.1%; B: PVDF@0.5%DNA; C: PVDF@5%DNA.

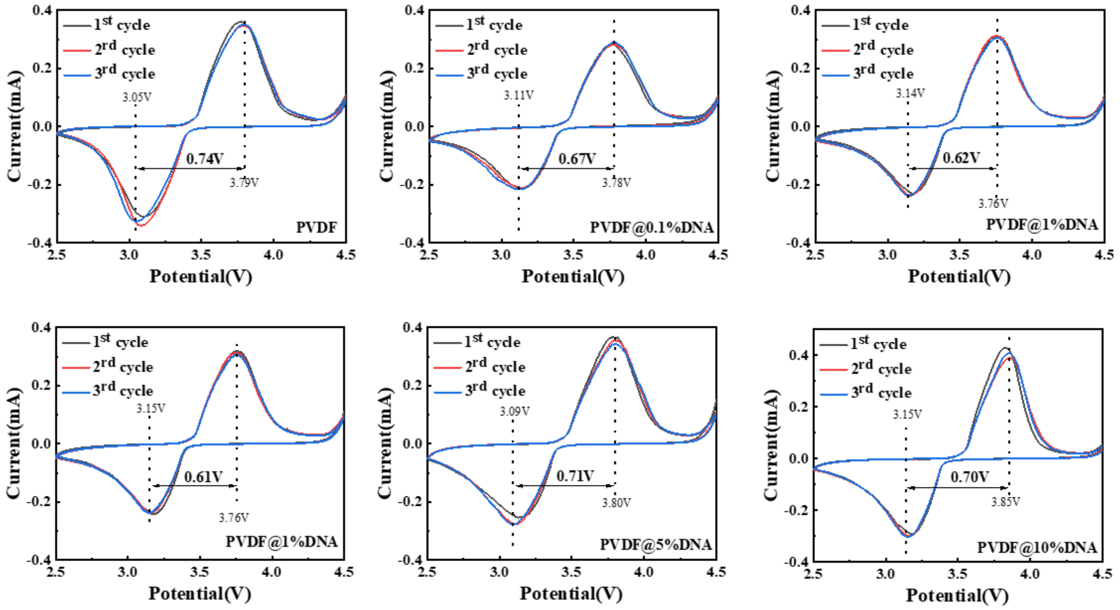

Figure S2. CV curves of solid polymer electrolytes.

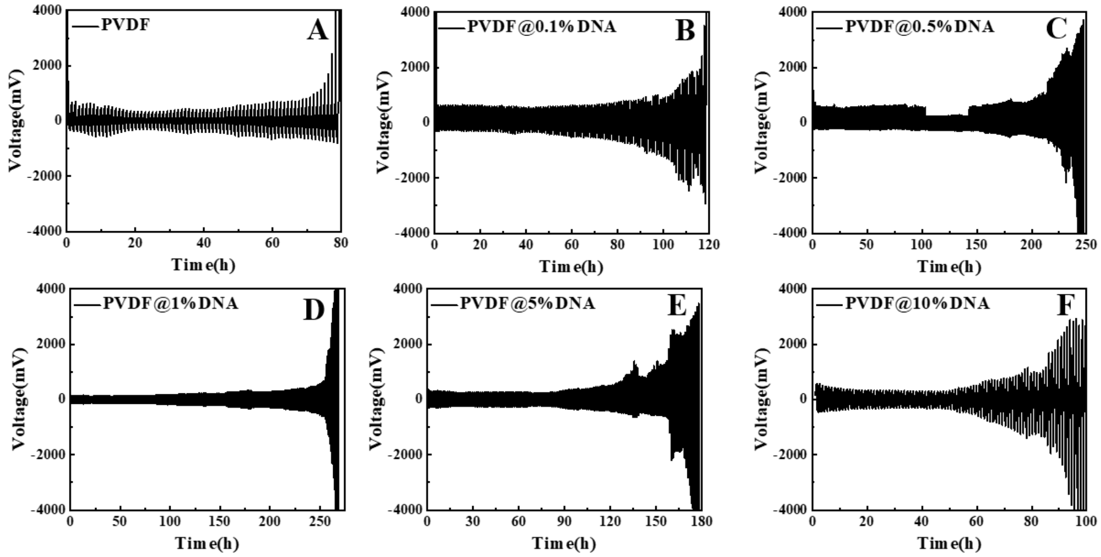

Figure S3. Galvanostatic cycling (C) of Li plating/stripping of Li/SPE/Li symmetrical batteries at room temperature (25℃), with 0.6 $mA\cdot cm^{-2}$ current density of solid polymer electrolytes.

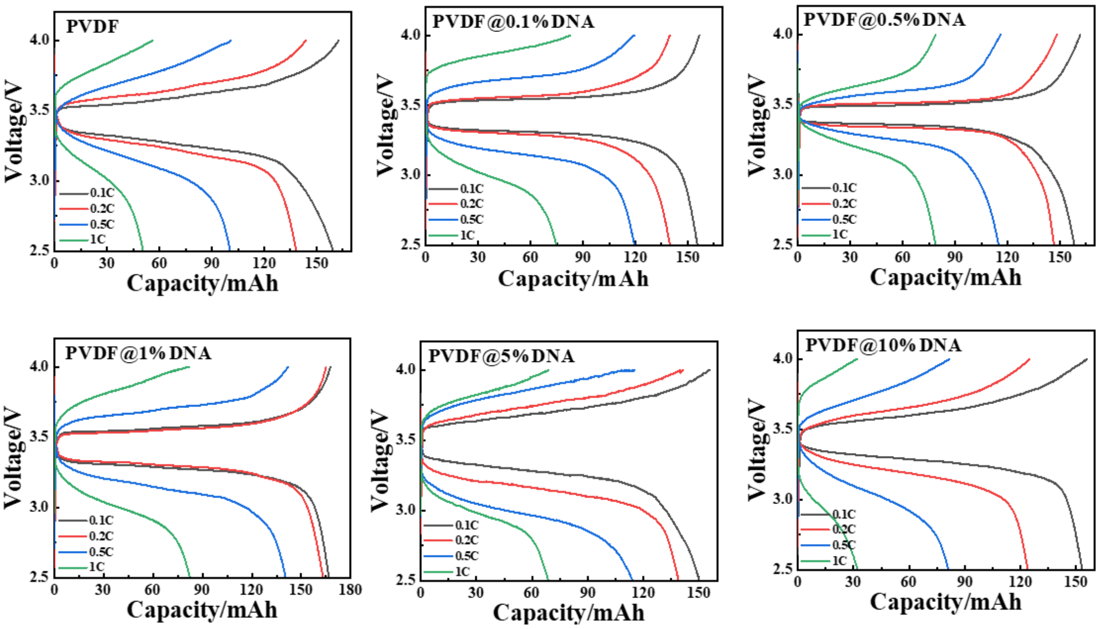

Figure S4. The charge-discharge curves for $LiFePO_4$/SPE/Li all-solid-state batteries under different C-rate.

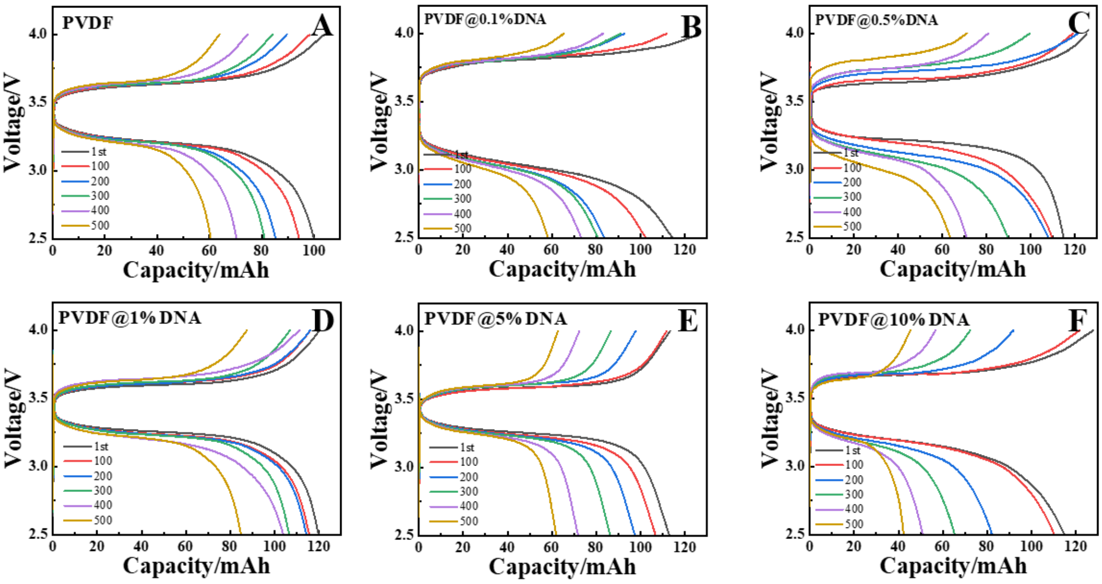

Figure S5. The capacity-voltage profiles for $LiFePO_4$/SPE/Li all-solid-state batteries under 0.5C.
